# Reducing Delays in Aortic Stenosis Treatment: A Clinical Audit of the Transcatheter Aortic Valve Implantation (TAVI) Pathway and Quality Improvement Initiatives

**DOI:** 10.7759/cureus.96731

**Published:** 2025-11-13

**Authors:** Orzo Shrestha, Honey Joshi, Amrit KC, Edney Boston-Griffiths, Mushbiq Manzoor

**Affiliations:** 1 Cardiology, Royal Bournemouth Hospital, Bournemouth, GBR; 2 Geriatrics, Poole General Hospital, Poole, GBR; 3 General Medicine, Dr. Iwamura Memorial Hospital, Bhaktapur, NPL; 4 Cardiology, Dorset County Hospital, Dorchester, GBR

**Keywords:** aortic stenosis (as), bcis standards, clinical audit system, nice ng208, transcatheter aortic valve implantation (tavi)

## Abstract

Background

Severe aortic stenosis (AS) is a prevalent and life-threatening condition in older adults. Timely valve replacement, either surgical or transcatheter, significantly improves survival. This audit evaluated our transcatheter aortic valve implantation (TAVI) pathway to identify delays and implement targeted quality improvements.

Methods

A retrospective audit of 29 consecutive patients with severe symptomatic AS referred for TAVI at a tertiary cardiac centre was conducted. Data on three key intervals, namely, referral-to-clinic, clinic-to-multidisciplinary team (MDT) decision, and MDT-to-procedure, were extracted from hospital records. Compliance with predefined timing targets (≤6 weeks per interval, ≤18 weeks total) was assessed, along with wait-list and 30-day mortality. Descriptive statistics summarized waiting times and compliance rates.

Results

The mean age was 76.3±9.8 with a median of 79; 18 (62%) were male, and 25 (86%) presented in New York Heart Association (NYHA) class III/IV heart failure. The mean total referral-to-TAVI time was 155±24 days (median 155), exceeding the 126-day benchmark; only three (10%) received treatment within 18 weeks. Referral-to-clinic targets were largely met in 23 (79%) patients, but major delays occurred between clinic and MDT (mean 56 days) and MDT-to-procedure (mean 64 days). Two (6.9%) patients died while awaiting TAVI, and among the 27 who underwent TAVI, one (3.7%) died within 30 days.

Conclusions

This audit revealed substantial delays in the TAVI pathway, primarily due to diagnostic and scheduling bottlenecks, resulting in prolonged waiting times and avoidable adverse outcomes. A quality improvement action plan, including a rapid-access valve clinic, one-stop investigations, increased procedural capacity, and active wait-list monitoring, has been implemented. Re-audit will evaluate the impact on reducing waiting times and improving patient outcomes.

## Introduction

Aortic stenosis (AS) is the most prevalent valvular heart disease in developed nations, particularly among the elderly. Population studies estimate that about 3.4% of individuals aged ≥75 years have severe AS [1]. Once symptoms such as exertional dyspnea, chest pain, or syncope appear, the prognosis is poor, approximately 50% mortality at two years and ~3% survival at five years, worse than many advanced cancers [2]. The disease often progresses to heart failure or sudden cardiac death, making valve replacement an urgent necessity once symptoms develop.

Transcatheter aortic valve implantation (TAVI) has transformed the management of severe AS, initially for high-risk surgical candidates and now extending to intermediate- and low-risk groups [3]. Multiple randomized trials confirm that TAVI is non-inferior or superior to surgical aortic valve replacement (SAVR) in suitable patients, offering improved survival and recovery with less invasiveness [4-9]. In the United Kingdom, over 5,000 TAVIs were performed in 2019 [2], yet this remains insufficient for rising demand. The burden of valvular heart disease is projected to double by 2050 [10].

A 2019 national TAVI survey showed that half of UK centres exceeded recommended timelines, with an average referral-to-procedure time of ~155 days, far beyond the 126-day (18-week) target [11]. Delays of six months have been linked to ~23% pre-procedure mortality [12,13]. Given that symptomatic severe AS represents a medical urgency, such delays can lead to preventable deaths and worsened outcomes.

To address this, the National Health Service (NHS) mandates an 18-week maximum referral-to-treatment interval for non-urgent care [14]. The British Cardiovascular Intervention Society (BCIS) formalized this standard for TAVI, targeting ≤6 weeks for each phase (referral-to-clinic, clinic-to-multidisciplinary team (MDT), and MDT-to-procedure) [9]. Similarly, the National Institute for Health and Care Excellence (NICE) NG208 stresses rapid specialist assessment (within two weeks) and prompt intervention for severe symptomatic valve disease [3].

Rationale for the audit

Our tertiary cardiac centre noted persistent post-pandemic delays in the TAVI pathway, spanning referral, diagnostic work-up, and procedural scheduling. Recognizing the clinical risk of such delays, this audit evaluated our performance against BCIS and NICE standards to identify bottlenecks and initiate targeted quality improvements. The ultimate goal was to ensure timely, life-saving treatment for patients with severe AS in alignment with national benchmarks.

## Materials and methods

Audit design and setting

This registered clinical audit was conducted at Dorset County Hospital, Dorchester, England, a tertiary cardiac centre in the United Kingdom. As a retrospective service evaluation, it did not require informed consent or NHS Research Ethics Committee review. Records of all patients referred for TAVI between January and December 2022 were reviewed. Twenty-nine consecutive patients with severe symptomatic AS who underwent TAVI or SAVR or had documented outcomes while waiting were included. The sample reflected the annual TAVI caseload and was adequate to identify systemic pathway delays.

Audit standards

Audit standards were derived from (1) the BCIS 2019 benchmark and (2) the NICE guideline NG208. BCIS recommends a total referral-to-treatment time of ≤18 weeks (126 days), subdivided into three intervals of ≤6 weeks each: referral-to-clinic, clinic-to-MDT decision, and MDT-to-procedure [9]. NICE similarly emphasizes timely specialist assessment and prompt intervention for severe valve disease, ideally within two weeks for symptomatic or high-risk patients [3]. For quantitative analysis, BCIS timing metrics were used as the principal standard, consistent with the NHS 18-week Referral to Treatment (RTT) policy, and results were contextually compared with published national data [2,11].

Data collection

Patient timelines were mapped using hospital databases (Solus, Change Healthcare, and digital patient records). Four key dates were extracted: (1) referral date, initial referral receipt or diagnostic trigger; (2) clinic assessment, first cardiology/valve clinic review; (3) MDT decision, date of heart team approval for TAVI; and (4) procedure date, date of TAVI or AVR. Three corresponding intervals were calculated: (A) referral-to-clinic, (B) clinic-to-MDT, and (C) MDT-to-procedure. Outcomes (death before or within 30 days after TAVI) were also noted. Data verification was performed independently by two audit leads, cross-checking discrepancies with source records and the TAVI coordinator. Demographic and clinical variables (age, sex, comorbidities, New York Heart Association (NYHA) class) were recorded for cohort characterization.

Statistical analysis

Descriptive analysis was performed using Microsoft Excel (Microsoft Corporation, Redmond, Washington, United States) and IBM SPSS Statistics for Windows, Version 26.0 (Released 2019; IBM Corp., Armonk, New York, United States). Mean±SD, median, and range were reported for each interval and compared with BCIS targets (≤42 days per phase, ≤126 days total). Compliance rates (%) meeting each benchmark were calculated. No inferential statistics were applied, as this was a quality improvement audit. Differences exceeding 14 days beyond targets were deemed clinically significant. Findings were reviewed by the heart valve team, leading to the development of a targeted action plan.

## Results

Baseline characteristics

The baseline characteristics of the 29 audited patients reflected a typical high-risk elderly population undergoing evaluation for aortic valve intervention. The mean age was 76.3±9.8 years (range 54-94), with 18 (62%) male patients. All patients had severe symptomatic AS, and 25 (86%) patients presented with advanced heart failure symptoms (NYHA class III or IV). Comorbid conditions were common: hypertension present in 18 (62%), coronary artery disease in 12 (41%), and diabetes mellitus in 10 (34%) were the most frequent, while chronic kidney disease and chronic obstructive pulmonary disease were present in six (21%) and five (17%) patients, respectively. The average predicted operative risk was moderate to high, with a mean Society of Thoracic Surgeons (STS) predicted risk of mortality (PROM) score of 5.8% and a EuroSCORE II of 6.5%, and nearly one-third of the cohort, nine (31%), were classified as high surgical risk or inoperable, making them suitable for TAVI. Most patients, 27 (93%), ultimately underwent TAVI, while two (7%) received SAVR. Despite these interventions, two (6.9%) patients died while awaiting the procedure, and among the 27 who underwent TAVI, one (3.7%) died within 30 days post-procedure, underscoring the vulnerability of this cohort and the potential impact of procedural delays on outcomes. Overall, the data highlighted the elderly, comorbidity-burdened population for whom timely intervention was critical to prevent adverse events and optimize survival (Table 1).

**Table 1:** Baseline characteristics of patients referred for aortic valve intervention (N=29) *30-day mortality calculated among patients who underwent TAVI (n=27). NYHA: New York Heart Association; MI: myocardial infarction; CABG: coronary artery bypass grafting; eGFR: estimated glomerular filtration rate; COPD: chronic obstructive pulmonary disease; TIA: transient ischemic attack; STS PROM: Society of Thoracic Surgeons predicted risk of mortality; TAVI: transcatheter aortic valve implantation; SAVR: surgical aortic valve replacement

Characteristic	Value/n (%) or mean±SD (range)
Demographics	
Age (years), mean±SD (range)	76.3±9.8 (54-94)
Median age (years)	79
Male	18 (62%)
Female	11 (38%)
Clinical characteristics	
Severe symptomatic aortic stenosis	29 (100%)
NYHA classes III-IV	25 (86%)
Diabetes mellitus	10 (34%)
Coronary artery disease (prior MI or CABG)	12 (41%)
Hypertension	18 (62%)
Chronic kidney disease (eGFR <60 ml/min)	6 (21%)
COPD	5 (17%)
Peripheral vascular disease	3 (10%)
Prior stroke or TIA	2 (7%)
Risk assessment	
STS PROM (predicted 30-day mortality, %)	5.8±2.5
Logistic EuroSCORE II (%)	6.5±2.0
High-surgical-risk/inoperable patients	9 (31%)
Intermediate-surgical-risk patients	20 (69%)
Procedural outcomes	
Underwent TAVI	27 (93%)
Underwent SAVR	2 (7%)
Death on the waiting list	2 (6.9%)
30-day post-procedure mortality	1 (3.7% of 27 TAVI patients)*

**Figure 1:**
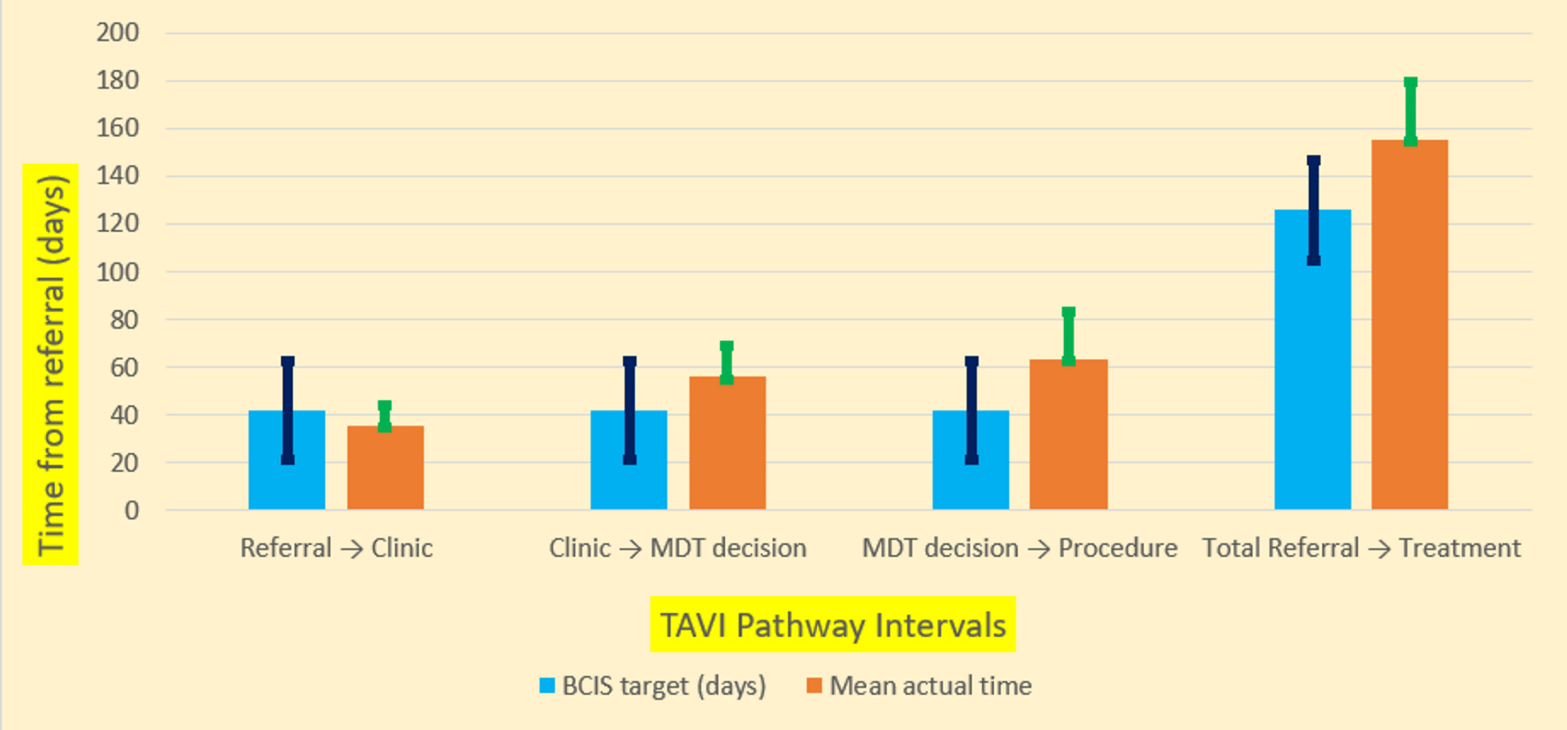
Mean waiting times versus target intervals in the TAVI referral-to-treatment pathway Each pair of bars shows the average time observed in our audit (orange, "Mean Actual") against the maximum recommended timeframe per BCIS guidelines (blue, "Target"). Error bars indicate one standard deviation. The first three intervals correspond to referral-to-clinic, clinic-to-MDT decision, and MDT-to-procedure; the fourth category represents total referral-to-treatment time. TAVI: transcatheter aortic valve implantation; BCIS: British Cardiovascular Intervention Society; MDT: multidisciplinary team

The audit demonstrated substantial delays in the TAVI referral-to-treatment pathway compared with BCIS benchmarks [9]. Figure 1 illustrates the mean waiting times versus target intervals, and Table 2 summarizes the detailed results.

**Table 2:** Waiting-time intervals vs. BCIS standards Targets per the BCIS 2019 guidelines [9]. Actual times expressed as mean±SD with median (range); compliance reported as n (%). BCIS: British Cardiovascular Intervention Society; MDT: multidisciplinary team

Pathway interval	BCIS target (days) [9]	Actual performance (mean±SD; median (range))	Compliance (% meeting target)
Referral → clinic	42 (6 weeks)	35.6±8.9; 34 (21-55)	23/29 (79%)
Clinic → MDT decision	42 (6 weeks)	56.1±13.1; 58 (30-84)	5/29 (17%)
MDT decision → procedure	42 (6 weeks)	63.7±20.0; 64 (28-94)	4/29 (14%)
Total referral → treatment	126 (18 weeks)	155.4±23.9; 153 (96-194)	3/29 (10%)

Interval A: referral-to-clinic

The mean time from referral to first valve clinic review was 35.6±8.9 days (median 34; range 21-55). Most patients were seen promptly, with 79% (23/29) meeting the six-week target and over half within four weeks. This indicated that referral triage and initial scheduling were generally efficient, although occasional communication lapses prolonged individual cases.

Interval B: clinic-to-MDT decision

Delays became evident after the clinic stage. The mean interval from clinic visit to MDT decision was 56.1±13.1 days (median 58; range 30-84), exceeding the 42-day target; only 17% (5/29) achieved compliance. Contributing factors included backlog in diagnostic imaging, particularly CT angiography, often delayed 4-8 weeks, limited MDT frequency, and pending ancillary assessments. Similar findings were reported in other UK centres, where average CT completion was 44 days and only 52% met the six-week goal [15].

Interval C: MDT decision-to-procedure

After the heart team decision, the mean wait to TAVI was 63.7±20.0 days (median 64; range 28-94). Compliance fell further, with only 14% (4/29) treated within six weeks. Most patients, 25 (86%), waited longer than eight weeks, largely due to limited procedural capacity approximately one dedicated TAVI list per week and occasional cancellations. This stage represented the single longest delay, highlighting a mismatch between demand and cath-lab availability.

Total referral-to-treatment time

Cumulatively, patients waited an average of 155.4±23.9 days (≈22 weeks) from referral to TAVI (median 153; range 96-194), surpassing the BCIS maximum of 126 days (18 weeks) [9]. Only three patients (10%) received treatment within the recommended timeframe. Even though early referral stages performed well, downstream investigation and scheduling delays caused nearly 90% of patients to breach the target. The mean delay beyond the benchmark was roughly 29 days, consistent with national data reporting an average 155-day TAVI wait and only 19% compliance [2].

Adverse outcomes

Two patients (6.9%) died while awaiting TAVI: one from decompensated heart failure three months post-MDT decision and another from sudden deterioration five months after referral. These findings align with previously reported 3.7-11.6% wait-list mortality for aortic valve interventions [13]. Among the 27 patients who underwent TAVI, 30-day mortality was 1 (3.7%), comparable to published outcomes for high-risk elderly cohorts. Most survivors, 26 (96%), had favourable short-term recovery, suggesting benefit once treated, but highlighting that prolonged waits likely worsened pre-procedural status.

Compliance summary

Only about six (21%) patients met all three BCIS interval standards, with the highest adherence in the referral-to-clinic stage, 23 (79%), and poorest in the later phases, five (17%) and four (14%), respectively. No elective case followed the ideal 18-week trajectory unless fast-tracked for urgency.

In summary, only three (10%) patients were treated within 18 weeks, with the greatest delays between MDT decision and TAVI. These findings confirmed a significant deviation from BCIS and NICE standards [3,9] and prompted the development of a targeted improvement plan to streamline investigations, expand procedural capacity, and reduce waiting-list mortality.

## Discussion

This audit demonstrated that our TAVI service delivered treatment for severe symptomatic AS significantly slower than recommended, with an average referral-to-procedure interval of approximately 22 weeks, exceeding the BCIS benchmark of 18 weeks for definitive treatment [9]. Only three (10%) patients received TAVI within this timeframe, with the main delays occurring during diagnostic work-up and procedural scheduling. Several patients clinically deteriorated, and two died while awaiting intervention.

These findings align with national data showing that prolonged TAVI waits are widespread across the United Kingdom. The 2019 UK TAVI Survey reported a median referral-to-procedure time of 141 days, with fewer than half of centres meeting the ≤18-week standard [14]. Similarly, Hewitson et al. identified diagnostic bottlenecks and limited procedural capacity as primary causes of delay, consistent with our local experience [2]. Nationally, waiting-list mortality remains a major concern, with an estimated 299 patients dying while awaiting TAVI in 2019, equating to over 500 potentially avoidable deaths per year [14].

The clinical impact of such delays is considerable. Once symptoms appear, severe AS carries a median survival of only 1-2 years, worse than many cancers [16]. Our observed waiting-list mortality of two (6.9%) patients parallels prior reports: Ali et al. estimated a 23% mortality with six-month TAVI delay [14], Poinsignon et al. reported 5.8% mortality among TAVI wait-listed patients [16], and Malaisrie et al. found 11.7% mortality at six months for SAVR candidates [13]. In our audit, both patients who died had already been accepted for TAVI but did not reach the procedure in time, underscoring that excessive waits have direct and preventable consequences.

Causes of delay

The principal bottleneck occurred in the diagnostic and MDT phase, where limited CT angiography capacity and fragmented scheduling prolonged the clinic-to-decision interval. Similar diagnostic backlogs have been cited as dominant contributors to TAVI pathway breaches nationally [2]. Sequential rather than parallel work-up processes also extended timelines. In the later phase, limited procedural capacity, only one TAVI list per week, further extended waits, consistent with the Valve for Life and BCIS reports highlighting cath-lab constraints as key limiting factors [11,14]. Referral inefficiencies and incomplete documentation from peripheral hospitals added further administrative delay, echoing findings from national audits [2].

Comparison with guidelines and literature

The BCIS 18-week standard [9] and NICE guideline NG208 [3] both emphasize timely treatment to improve survival and quality of life. However, as Ali et al. noted, UK TAVI provision still lags behind European standards, with structural barriers leading to "excess mortality" [14]. Encouragingly, several UK quality improvement studies, such as Time to TAVI: Streamlining the Pathway, demonstrated that adopting one-stop clinics and local pre-assessment reduced average referral-to-TAVI time to ~90 days without adverse outcomes [2]. These data informed our local improvement plan.

Limitations

This was a single-centre, retrospective audit with a small sample size (n=29). While findings may not be statistically generalizable, they are consistent with national trends. Minor date discrepancies cannot be excluded, though cross-verification minimized error. The audit focused on timelines rather than detailed risk-outcome analysis or cost implications, which will be addressed in future cycles.

Action plan

Based on these findings, our centre implemented four key interventions. First, a rapid-access valve clinic was introduced, allowing direct referral from echocardiography to expedite assessment. Second, a one-stop diagnostic work-up was introduced so that imaging, laboratory tests, and multidisciplinary review could be completed in parallel. Third, TAVI capacity was increased by adding additional cath-lab sessions and implementing real-time wait-list monitoring. Lastly, an urgent case policy was adopted with plans for ongoing re-audit to ensure that high-risk patients receive treatment within four weeks.

These initiatives mirror the Valve for Life recommendations [14] and aim to raise 18-week compliance from 3/29 (10%) to at least 23/29 (80%) within one year. Early follow-up will track both waiting-time reduction and outcome improvement.

## Conclusions

This clinical audit revealed substantial delays in the referral-to-treatment pathway for TAVI at our centre, with most patients waiting well beyond the BCIS's 18-week target. These delays were associated with preventable adverse outcomes, including wait-list mortality, highlighting the urgency of timely intervention in severe AS.

By pinpointing the major bottlenecks, diagnostic work-up and procedural scheduling, the audit catalyzed meaningful service redesign. A targeted action plan has now been implemented, featuring a rapid-access valve clinic, one-stop investigations, increased TAVI capacity, and active wait-list monitoring. These measures are expected to markedly reduce waiting times and align our performance with national standards.

Timely referral-to-TAVI not only fulfils a performance metric but directly improves outcomes: fewer patients deteriorate or die while waiting, symptoms are relieved sooner, and overall survival and quality of life improve. Experiences from other UK centres and our early data suggest such improvements are achievable.

This audit also underscores the power of clinical audit as a driver of quality improvement. Through continuous audit-improve-reaudit cycles, we aim to sustain progress and share best practices with other centres. With streamlined pathways and adherence to BCIS and NICE guidance, patients with severe AS can receive lifesaving treatment without unnecessary delay.
